# Melanoma Extension for Community Healthcare Outcomes: A Feasibility Study of Melanoma Screening Implementation in Primary Care Settings

**DOI:** 10.7759/cureus.15322

**Published:** 2021-05-29

**Authors:** Mirna Becevic, Emily Smith, Mojgan Golzy, Ramakrishna Bysani, Adam Rosenfeld, Ellen R Mutrux, Kimberly Hoffman, Emmanuelle Wallach, Jane A McElroy, Karen Edison

**Affiliations:** 1 Dermatology, University of Missouri School of Medicine, Columbia, USA; 2 Health Management and Informatics, University of Missouri School of Medicine, Columbia, USA; 3 Missouri Telehealth Network, University of Missouri School of Medicine, Columbia, USA; 4 Family Medicine, University of Missouri School of Medicine, Columbia, USA

**Keywords:** melanoma diagnosis, early screening, continuing health education, virtual learning, collaborative learning

## Abstract

Introduction

Melanoma incidence rates are rising faster than the rates of any other malignancy. As a major global public health concern, melanoma can be identified by a visual exam not requiring expensive invasive procedures. However, non-dermatologists lack specialized training and skills to identify high-risk patients and implement melanoma skin screenings during regular exams. Most patients from rural and underserved areas have inadequate access to specialty dermatologic care, which can potentially lead to later-stage melanomas and poor patient outcomes. The objective of this study was to identify facilitators and barriers to the implementation of risk surveys and melanoma skin screenings in primary care settings through live interactive education and the telementoring project - Melanoma ECHO (Extension for Community Healthcare Outcomes).

Methods

This cross-sectional study was designed with theoretical concepts from dissemination and implementation research. Monthly Melanoma ECHO sessions were integrated into an ongoing Dermatology ECHO at the University of Missouri, Columbia, Missouri, USA, from April 2018 to February 2019. Ten primary care providers, medical doctors/doctors of osteopathic medicine (MDs/DOs), nurse practitioners (NPs), and physician assistants (PAs), from across Missouri participated. Eleven virtual monthly melanoma-related didactics and case-based discussions were provided to participants. Information regarding risk factors, risk surveys, and screening techniques was provided. Ongoing telementoring and guidance were also provided for de-identified real-life patient cases. The main outcomes and measures of the study were to identify the facilitators and barriers of risk survey and melanoma skin screenings in primary care settings and to quantify the number of high-risk patients identified by participating providers and the number of new melanomas detected by visual exams during the study period.

Results

The primary reason why six out of 10 providers reported participation in Melanoma ECHO was that implementing melanoma skin screenings in their practice was made easier as it increased their confidence. Nine providers reported increased knowledge, and eight cited professional networking as other facilitators. The main perceived barrier to melanoma skin screening was lack of administrative and nursing support, and six providers indicated that lack of time to incorporate skin exams was also a barrier. Combined, ten participants reported identifying 976 high-risk patients during the study period and detecting 36 new melanomas.

Discussion and conclusion

Our findings indicate that primary care providers may benefit from attending regularly scheduled and focused specialized telementoring sessions, such as Melanoma ECHO. Ongoing support from specialists may help providers practicing in rural and isolated areas with the successful integration of risk surveys and melanoma skin screenings in primary care settings. Further Melanoma ECHO sessions with a more diverse group of primary care providers are needed to better understand the generalizability of the results.

## Introduction

The incidence rates of melanoma, the most deadly of all skin cancers, have been rising faster than the rates of any other malignancy [1]. The latest estimate indicates that over 100,000 people will be diagnosed with melanoma in 2021 in the United States (US) [2]. Melanoma is also the most commonly diagnosed cancer in 25- to 29-year olds in the United States, where one person dies of melanoma every hour, every day [3].

If detected and diagnosed early, melanoma can often be cured [4]. Unlike many other cancers that require invasive and expensive procedures for detection, such as colon or breast cancers, most melanomas can be detected by a visual exam. In several studies, a full body examination by trained dermatologists led to the identification of early-stage and thinner melanomas, improved the chance of survival, and had better patient outcomes [5,6].

Lack of timely access to qualified dermatologists, however, is a significant barrier for many patients living in rural, isolated, and underserved areas. Most dermatologists practice in highly populated urban areas, resulting in an unevenly distributed workforce. Poor access to care produces astounding disparities in melanoma survival rates. A recent study by Hopkins et al. found that melanoma survival rates are positively associated with a higher density of practicing dermatologists [7-9]. With only 117 dermatology residency programs in the country graduating less than 500 dermatologists every year, it is clear that the shortage of dermatologists will not improve in the near future [9,10]. Since the burden of melanoma and other skin cancers has been increasing, it is not surprising that many patients with skin concerns seek care with non-dermatologists [11,12].

There is growing literature that investigates supplementing workforce shortages and undersupply of dermatologists with non-dermatologist clinicians [9]. These articles can generally be categorized into two separate groups that study (a) experience with care and (b) diagnostic concordance.

(a) Experience with care: A population health study found that patients seen by non-dermatologists reported their clinicians had poorer knowledge regarding melanoma and provided fewer therapy options and less information regarding their diagnosis [13,14]. Spinks et al. analyzed patient preferences regarding teledermoscopy screening to detect early melanoma and found that patients preferred skin screening by clinicians rather than self-examination [15]. They also reported that patients preferred for their screening results to be reviewed by dermatologists because of increased diagnostic accuracy and to avoid unnecessary biopsies, which are more commonly performed by non-dermatologists [15,16].

(b) Diagnostic concordance: Several studies reported that primary care physicians, when compared to dermatologists, have limited skills regarding melanoma detection [17,18]. In addition, patients with skin problems are referred to dermatologists at a higher rate than patients with non-dermatologic conditions to other specialties [19,20].

A systematic review of skin cancer education for primary care physicians found that most medical schools provide only limited experience in dermatology, causing barriers such as poor self-efficacy and diagnostic skills for many practicing primary care providers (PCPs) [19]. However, interest in melanoma detection is increasing among PCPs, and there are many isolated interventions with different educational techniques available [19]. Unfortunately, they lack a standardized approach, making them hard to evaluate and compare [19]. Out of the 13 interventions analyzed by Goulart et al., only six used an interactive delivery mechanism, whereas others used literature, feedback, and additional web-based formats [19].

Our study was based on a novel virtual collaborative knowledge-sharing network - Melanoma ECHO (Extension for Community Healthcare Outcomes) - aimed at providing ongoing telementoring to participating PCPs during the study period. In order to expand knowledge regarding perceived facilitators and barriers of melanoma skin screening in primary care settings for PCPs participating in Melanoma ECHO, we completed a feasibility study. Our study had two aims: (1) to explore the perceptions of participating PCPs regarding implementation of risk survey and melanoma skin screenings in their clinics and (2) to quantify the number of high-risk patients screened and new melanomas detected by participating PCPs.

Preliminary findings were previously abstracted as a meeting poster at the 2019 MetaECHO conference held on March 13-16, 2019 in Albuquerque, New Mexico.

## Materials and methods

The University of Missouri Institutional Review Board's approval was obtained prior to the study.

Study framework

Our study was designed with key theoretical concepts from guideline dissemination and implementation research [21], focusing specifically on (1) interactive and participatory educational interventions and (2) behavior change and promoting ongoing maintenance of change, influenced by environment and social support.

Interactive and Participatory Educational Intervention: Melanoma ECHO

The University of Missouri (MU), Missouri Telehealth Network (MTN), and the MU Department of Dermatology implemented the first Dermatology ECHO program in the United States in 2015. The program aims to create a virtual learning and knowledge-sharing network for PCPs in Missouri and to provide telementoring using a case-based learning approach. Dermatology ECHO is comprised of a hub team of dermatologists, pediatric dermatologists, and dermatopathologists who meet via live interactive video with a number of participating PCPs from across the state to provide continuing education, guidance, and mentoring regarding the PCPs’ real-life patient cases.

In 2018, we developed a Melanoma ECHO, which was held monthly via live interactive video sessions during the 11 months of the study period. The need for Melanoma ECHO arose from discussions in the general Dermatology ECHO. The goal of Melanoma ECHO was to provide tailored didactics and create a non-judgmental environment for participating PCPs to discuss their de-identified cases. The Melanoma ECHO hub team consisted of dermatopathologists, general dermatologists, pediatric dermatologists, and nurse practitioners (NPs) from a dermatology clinic.

Behavior Change and Promoting Ongoing Maintenance of Change, Influenced by Environment and Social Support: Telementoring Approach

Melanoma ECHO was developed in the context of adult learning theory and active learning in medical education. This framework provided robust bases for the development of didactic topics and discussions, based upon the evidence on how adults learn [22]. More specifically, each Melanoma ECHO session was rooted in a transformative learning theory approach, which focused on the “cognitive process of meaning-making” [22]. This was achieved through the “learning cycle”: (a) Participating PCPs’ gained melanoma-related experience by attending virtual collaborative knowledge-sharing ECHO sessions. (b) Participating PCPs shared their de-identified real-life patient cases for mentoring and education. Participating PCPs and hub-team experts all added to an increased learning experience by reflecting and sharing their knowledge and opinions. (c) Participating PCPs shared their knowledge and expertise regarding other similar cases. (d) Participating PCPs applied newly gained knowledge and expertise to patients in their practice. (e) Participating PCPs continued attending ECHO sessions and screening patients in their clinic.

Melanoma ECHO sessions were dedicated to more than just adding new information in didactic presentations; they provided practical guided concepts that had the potential to change attitudes, perspectives, and behaviors of participating PCPs [22]. De-identified patient case presentations are used for telementoring and education, where the hub-team specialists demonstrate diagnostics and treatment approaches in addition to inviting participants to discuss and share their knowledge and opinions about other similar patient cases. The expert hub team emphasized an “all teach and all learn” non-judgmental environment supportive of sharing resources and knowledge among all learners [23].

Multiple data sources were used to validate the findings: (a) Attendance data was collected in iECHO, then exported and analyzed in Excel; (b) field notes from observational clinical data were written contemporaneously, then shared and discussed with the project principal investigator (PI); and (c) survey data was collected in REDCap (Vanderbilt University, Nashville, Tennessee, USA), also exported and analyzed in Excel.

Participants

We recruited a convenience sample of 15 PCPs from 11 different clinics: six medical doctors (MDs), two physician assistants (PAs), three doctors of osteopathic medicine (DOs), two family nurse practitioners (FNPs), one doctor of nursing practice (DNP), and one nurse practitioner (NP).

Inclusion criteria were PCP credentials (MD, DO, NP, or PA) and practicing primary care in Missouri, including family medicine, internal medicine, or obstetrics, gynecology, and women’s health. Eligible PCPs were approached by the project PI by email and phone, and written consent was received. Participating PCPs received monetary incentives for participation: $50 upon recruitment (month 1) and $100 upon completion of the final survey (month 11). The project PI was not a member of the Melanoma ECHO hub team.

Five PCPs withdrew from the study, citing lack of research time availability and increased clinic demands. Participating PCPs were asked to attend Melanoma ECHO sessions, offer melanoma risk surveys, and provide skin screenings to high-risk patients during the study period. Participating PCPs completed a 12-question survey upon completion of the study. They were also asked to report any melanomas newly diagnosed in their patient population during the course of the study.

Melanoma risk survey

Copies of the validated survey, Self-Assessment of Melanoma Risk Score (SAMScore), were provided to participating PCPs [24,25]. Quéreux et al. assessed the efficiency of the SAMScore and found that targeted screening using this score allows for 11 times fewer patient screenings than with a nontargeted screening [26]. This would allow non-dermatologists to screen primarily those that are identified as high-risk patients by the SAMScore questionnaire.

In accordance with the SAMScore survey, patients are identified as high-risk if they satisfy at least one of the following three criteria:

(A) Presence of at least three risk factors among the seven following risk factors: (1) skin type I (very fair skin, blonde or red hair, light eyes - blue or green, never tan, and always sunburn after sun exposure) or skin type II (fair skin, blonde or light brown hair, light eyes - blue or green, and usually sunburn), (2) freckling tendency, (3) number of melanocytic nevi > 20 on both arms, (4) severe sunburn during childhood or teenage years, (5) life in a country at low latitude, (6) a history of previous melanoma, (7) a history of melanoma in a first-degree relative,

(B) A subject under 60 years of age and a number of melanocytic nevi > 20 on both arms,

(C) A subject of 60 years old or over and a freckling tendency [26].

Clinic observations

One second-year medical student (RB) was recruited to observe the implementation of risk-screening questionnaires and skin screenings with seven participating providers in four different clinics. The student attended Dermatology and Melanoma ECHO sessions as an observer to become familiar with the knowledge-sharing model and didactics. The student met with the project PI to learn about the clinic observation structure and expectations. Clinics were selected based on the geolocation and proximity to the University of Missouri Health Center (UMHS) to avoid overnight expenses and other extended travel-related costs. Clinic visits were performed during months three to five of the study period. Medical students reported observations after each clinic visit during in-person meetings and with written reports describing perceived facilitators and barriers of the observed implementation processes.

Participating primary care providers' survey instrument

Since a validated survey of perceived facilitators and barriers of melanoma skin screening for PCPs attending virtual collaborative knowledge-sharing networks is not available, we developed a new 12-question survey (Appendix). The survey consisted of seven questions with multiple available answers, “check all that apply” (questions 1-7), four questions with free text answers (questions 8-11), and one question with a 0 to 100 sliding scale (question 12).

We completed a cognitive, content, and construct survey pilot. Four clinicians and one healthcare administrator not involved in the feasibility study reviewed the survey questions for content and grammatical accuracy. They also completed the preliminary testing to ensure the clarity and the order of the questions and were given the opportunity to provide suggestions for possible improvements. The survey instrument was finalized using validation testing recommendations.

## Results

Eleven experts continuing medical education (CME)-approved didactics were presented by a general dermatologist and dermatopathologist, and one pediatric melanoma lecture was presented by a pediatric dermatologist during the study period (Table 1).

**Table 1:** Melanoma ECHO didactic presentations ECHO, Extension for Community Healthcare Outcomes.

Month	Topic
Month 1	Introduction to Melanoma ECHO project skin screening demo
Month 2	Melanoma epidemiology, trends, and risks
Month 3	Melanoma diagnosis
Month 4	Patient education
Month 5	Melanoma staging and management
Month 6	Melanoma systemic treatments
Month 7	Dermatopathology of melanoma
Month 8	Melanoma mimics
Month 9	Pediatric melanoma
Month 10	Non-cutaneous melanoma and melanoma in skin of color
Month 11	Genetics of melanoma

As of May 1, 2020, the Dermatology ECHO has provided 140 live interactive sessions and 135 didactic presentations, and a total of 500 real-life de-identified patient cases have been discussed. There have been 285 unique attendees and 2,137 total contact hours since the inception of Dermatology ECHO (Table 2).

**Table 2:** Dermatology ECHO aggregate data ECHO, Extension for Community Healthcare Outcomes.

Date	Nov 20, 2015–May 1, 2020
Number of live interactive sessions	140
Number of didactic presentations	135
Number of real-life de-identified patient case discussions	500
Number of unique learners	285
Total number of learners (total contact hours)	2,137

For Melanoma ECHO, we recruited PCPs from different parts of the state. Table 3 provides the distribution of the PCP population, expected number, variance, and the 95% confidence interval (CI) for the sample’s count under the normal approximation and actual sample count.

**Table 3:** Distribution of recruited PCPs PCPs, Primary care providers; MDs, medical doctors; NPs, nurse practitioners; PAs, physician assistants.

	Population (n = 10,510)						Sample (n = 15)
PCP	n	%	E(np)	V(np)	95% CI for E(np)		n
MDs	5,000	0.476	7.140	3.730	3.350	10.920	9
NPs	4,000	0.381	5.715	3.532	2.030	9.400	4
PAs	1,510	0.144	2.160	1.846	-0.400	4.820	2

Actual sample counts are inside the CI of the expected sample counts, and so our sample is representative of the population.

The participating PCP survey data were analyzed using quantitative and qualitative methods. The chi-square test of independence was used to test the independency of the questions in the participating PCP survey. The Cramer’s V coefficient of association was used to measure the association between the items in different questions.

There was a negative nonsignificant association between items in question 1 “Which of the following made it easier to use the patient melanoma risk survey in your practice” (answer choices: administrative and nursing support in handing out surveys to patients; easy integration of surveys during the check-in process; the survey was easy to understand and use) and similar items in question 2 “Which of the following made it difficult to use the patient melanoma risk survey in your practice” (answer choices: lack of administrative and nursing support in handing out surveys to patients; surveys were difficult to integrate during the check-in process; the survey was difficult to use; other). For example, the coefficient of association was -0.41 between the administrative and nursing support in handing out the survey to patients (item 1 in question 1) and the lack of administrative and nursing support in handing out the survey to patients (item 1 in question 2). The p-value for the corresponding chi-square test was 0.52 indicating the independency of these items (Table 4).

**Table 4:** Melanoma ECHO contingency table of administrative support (Q1) by lack of administrative support (Q2) ECHO, Extension for Community Healthcare Outcomes.

Admin support	Lack of admin supp.		
	0	1	Total
0	2	3	5
	20	30	50
	40	60	
	33.33	75	
1	4	1	5
	40	10	50
	80	20	
	66.67	25	
Total	6	4	10
	60	40	100

We also found no significant association between items in question 3 “Which of the following made it easier to implement skin screenings in your practice” (answer choices: high-risk patients were in a gown and ready to skin exam; Melanoma ECHO didactics provided tips and tools that were helpful for implementation of skin screenings; participating in Melanoma ECHO increased my confidence in providing melanoma skin exams; patient readiness; and other) and items in question 4 “What were the barriers to skin screening implementation in your practice” (answer choices: It was hard to find time to integrate skin exams into regular appointments; Melanoma ECHO didactics did not provide the practical tips and tools I expected; patients refused full skin exams; and other). For example, the coefficient of association was -0.35 between the patient readiness and patient refused full skin exam (the p-value = 0.5) (Table 5).

**Table 5:** Melanoma ECHO contingency table of high-risk patients ready (Q3) by hard to find time to integrate skin exams (Q4) ECHO, Extension for Community Healthcare Outcomes.

High-risk patients ready for a skin exam	Hard to find time to integrate skin exams		
	0	1	Total
0	2	5	7
	20	50	70
	28.57	71.43	
	50	83.33	
1	2	1	3
	20	10	30
	66.67	33.33	
	50	16.67	
Total	4	6	10
	40	60	100

Participating PCP reported identifying 976 high-risk patients during the study period and detecting 36 new melanomas by visual exams. We did not have access to any patient identifiable data, including their counties. However, if we consider 36 newly diagnosed melanomas in a county with 180,000 population where the study academic medical center is located, then the melanoma incidence rate in our study is about 20 per 100,000, which is comparable with the overall melanoma incidence rate of 21.8 per 100,000 [27].

Perceived facilitators

Administrative and nursing support were indicated as the main facilitators to risk survey implementation in the primary care clinic setting in addition to easy survey integration during the check-in process and ease of use of the risk survey. Sixty percent of participating PCPs (6/10) reported that participating in Melanoma ECHO was the primary reason that made it easier for them to implement melanoma skin screenings in their practice because it increased their confidence in providing melanoma skin exams. Fifty percent (5/10) of participating PCPs reported patient readiness as another facilitator in addition to Melanoma ECHO didactics, tips, and tools (3/10).

Participating PCPs (9/10) reported that attending monthly Melanoma ECHO sessions increased their knowledge and that they appreciated professional networking (8/10) as well as collaborative melanoma discussions (7/10).

Participating PCPs found didactic topics on skin screening (7/10), melanoma diagnosis (7/10), and melanoma staging and management (5/10) most helpful. Only five participating PCPs responded to the question regarding the least helpful topic, which was indicated as the genetics of melanoma.

One participating PCP reflected on the ways in which Melanoma ECHO tele-education and mentoring were helpful during the project period: “ECHO Staff was always available and MOST helpful!!”

A total of five participating PCPs provided responses to the question “Is there something that could be done differently to assist primary care providers in identifying and screening high-risk melanoma patients?" (Table 6).

**Table 6:** Melanoma ECHO participating PCPs' comments and suggestions regarding perceived barriers ECHO, Extension for Community Healthcare Outcomes; PCPs, primary care providers.

Is there something that could be done differently to assist primary care providers in identifying and screening high-risk melanoma patients?	Other barriers to skin screening implementation in the primary care setting
“Get support of administration and nursing in all pointing out the importance of this.”	“Lack of confidence"
“Continue this plan. I 100% enjoyed the ECHO, but our topics were everything. For a new to derm that was overwhelming... I appreciated the help because everything comes up... but if I only did melanoma focus and melanoma ECHO education, I think it may have been more fruitful to me personally.”	"Fear of missing something"
“Expand the program to all PCP's in MO.”	"The patient falsely assured exam was completed.”
“Dermatoscope training (intense).”	“Time constraints”
“More time with patients.”	

Perceived barriers

The main barriers to the implementation of risk surveys in primary care clinic settings were a lack of administrative and nursing support as well as difficult integration of risk surveys during the patient check-in process. Participating PCPs (6/10) reported that finding time to incorporate skin exams into regular clinic visits was also a barrier, as was lack of confidence.

It was also noted during clinic observations that some patients had difficulties identifying their skin type on the risk survey. Anecdotal responses from patients indicated that a photo with an example of the skin type may make skin-type identification easier. SAMScore risk survey provides six different skin type options for patients with no visual aids: skin type I - very fair skin, blonde or red hair, light eyes (blue or green), never tan, and always sunburn after sun exposure; skin type II - fair skin, blonde or light brown hair, light eyes (blue or green), and usually sunburn; skin type III - dark skin, brown hair, and light to medium eye color; skin type IV - olive skin, dark brown hair, and brown eyes; skin type V - brown skin black hair, and black eyes; skin type VI - black skin, back hair, and black eyes [26]. Answering these questions correctly helps their providers identify if they are at high risk for melanoma.

Clinics also reported having non-English speaking patients and requested the SAMScore survey be translated into Spanish. The survey was subsequently translated and distributed to requesting participating PCPs.

## Discussion

Rural and underserved patients often delay or do not seek specialty dermatologic care due to travel distance and other constraints. Maldistribution of dermatologists is a growing concern affecting many rural Americans. For those who are unable to seek specialty care, timely melanoma skin exams in primary care settings may save lives [28]. Early detection of melanoma through skin screenings of high-risk patients results in an average five-year survival rate of over 98% for localized disease [28,29]. However, skin examination is typically not a part of a complete physical examination by PCPs, and only 8% of patients receive a full skin examination during their annual well visits. PCPs often cite lack of training, knowledge, and confidence in the diagnosis and treatment of skin cancer, further complicating the issue [28-30]. The purpose of our study was to provide ongoing telementoring and create a virtual network for participating PCPs to support them through the implementation of risk surveys and skin screenings in their daily practice.

Our study did not find a significant association between facilitators and barriers reported in the participating PCP survey regarding survey administration or implementation of skin exams. Anecdotal participating PCP evidence, however, suggested that expansion of Melanoma ECHO to other PCPs would promote learning and improve melanoma screening.

The main strengths of our study were reports from a real-life implementation study and an ongoing peer-support network. Our study had several limitations. The study was conducted in one primarily rural state, and further research needs to be conducted in different settings. Our study population consisted of a small number of practicing PCPs. We recommend further studies to include more participants from a more diverse group of PCPs.

## Conclusions

Implementation of risk questionnaires and skin screenings in primary care settings depend on several factors: time availability, staff support, but most importantly guidance and mentoring from dermatologists. As Melanoma ECHO programs spread to other states and countries, hopefully more PCPs will be trained to do skin screenings for melanoma in their high-risk patients. Other factors, such as patient readiness, and PCPs’ motivation are likely to have an influence as well.

## Appendices

Melanoma ECHO participating PCP survey

Please complete the survey below.

Thank you!

1. Which of the following made it easier to use the patient melanoma risk survey in your practice (select all that apply)?

Administrative and nursing support in handing out surveys to patients;

Easy integration of surveys during the check-in process;

Survey was easy to understand and use;

Other.

If other, please specify:

2. Which of the following made it difficult to use of patient melanoma risk survey in your practice?

Lack of administrative and nursing support in handing out surveys to patients;

Surveys were difficult to integrate during the check-in process;

Survey was difficult to understand;

Other.

If other, please specify:

3. Which of the following made it easier to implement skin screenings in your practice?

High-risk patients were in a gown and ready to skin exam;

Melanoma ECHO didactics provided tips and tools that were helpful for the implementation of skin screenings;

Participating in Melanoma ECHO increased my confidence in providing melanoma skin exams;

Patient readiness;

Other.

If other, please specify:

4. What were the barriers to skin screening implementation in your practice (select all that apply)?

It was hard to find time to integrate skin exams into regular appointments;

Melanoma ECHO didactics did not provide the practical tips and tools I expected;

Patients refused full skin exams;

Other.

If other, please specify:

5. In which ways were Melanoma ECHO tele-education and mentoring helpful during the project period (select all that apply)?

Monthly Melanoma ECHO sessions increased my knowledge and expertise;

Melanoma case presentations provided collaborative learning discussions;

I appreciated professional networking during Melanoma ECHO sessions;

Other.

If other, please specify:

6. Which of the following didactic topics were most helpful (check all that apply)?

Introduction to Missouri Foundation for Health (MFFH) research project;

Skin screening demo;

Melanoma epidemiology, trends, and risks;

Melanoma diagnosis;

Patient education;

Melanoma staging and management;

Melanoma systemic treatments;

Dermatopathology of melanoma;

Melanoma mimics;

Pediatric melanoma;

Non-cutaneous melanoma;

Melanoma in skin of color;

Genetics of melanoma.

7. Which of the following didactic topics were least helpful (check all that apply)?

Introduction to MFFH research project Skin screening demo;

Melanoma epidemiology, trends, and risks;

Melanoma diagnosis;

Patient education;

Melanoma staging and management;

Melanoma systemic treatments;

Dermatopathology of melanoma;

Melanoma mimics;

Pediatric melanoma;

Non-cutaneous melanoma;

Melanoma in skin of color;

Genetics of melanoma.

8. Are there melanoma-related topics we did not address? Yes but would have been helpful? No?

If yes, please specify:

9. Is there something that could be done differently to assist primary care providers in identifying and screening high-risk melanoma patients?

10. How many high-risk patients did you identify in your practice from April 2018 until March 2019?

11. How many new cases of melanoma did you diagnose from April 2018 until March 2019?

12. On a scale of 0 to 100, 0 being not likely and 100 being extremely likely, how likely are you to continue to use the high-risk patient survey and provide skin screening to high-risk patients in the future?

0                                              50                                             100

(Place a mark on the scale above)
